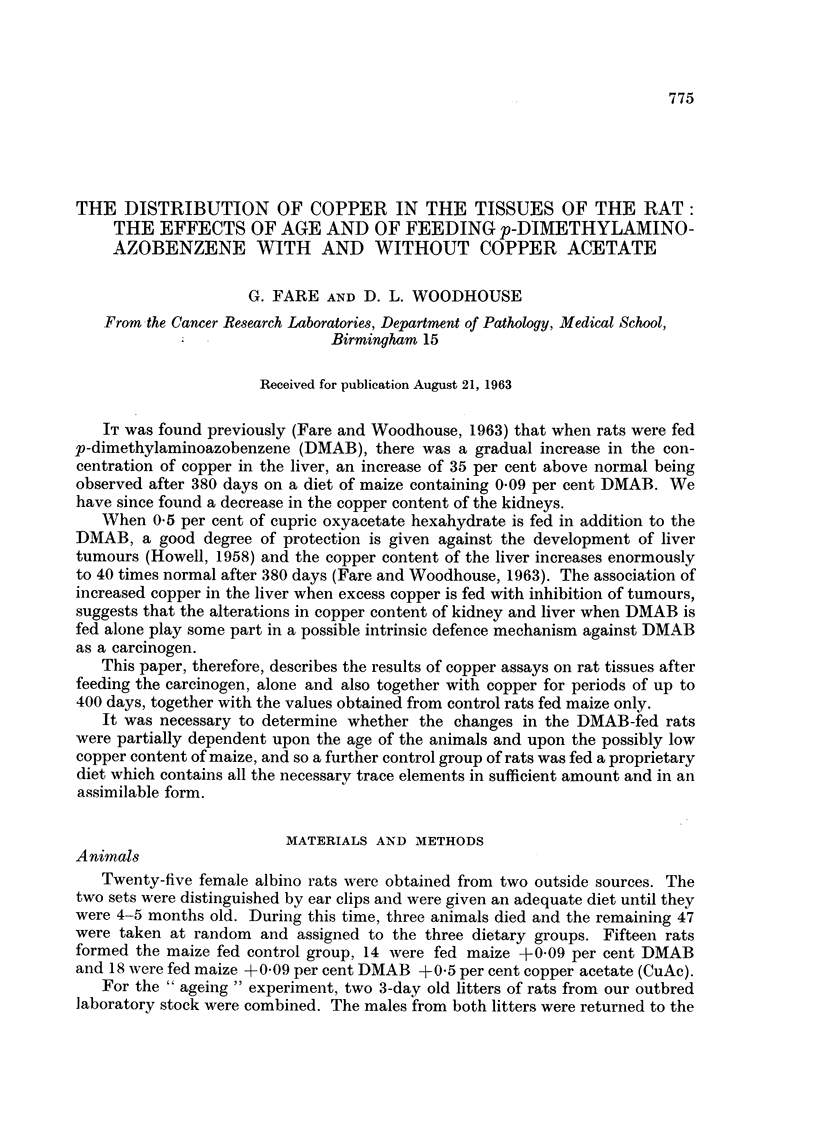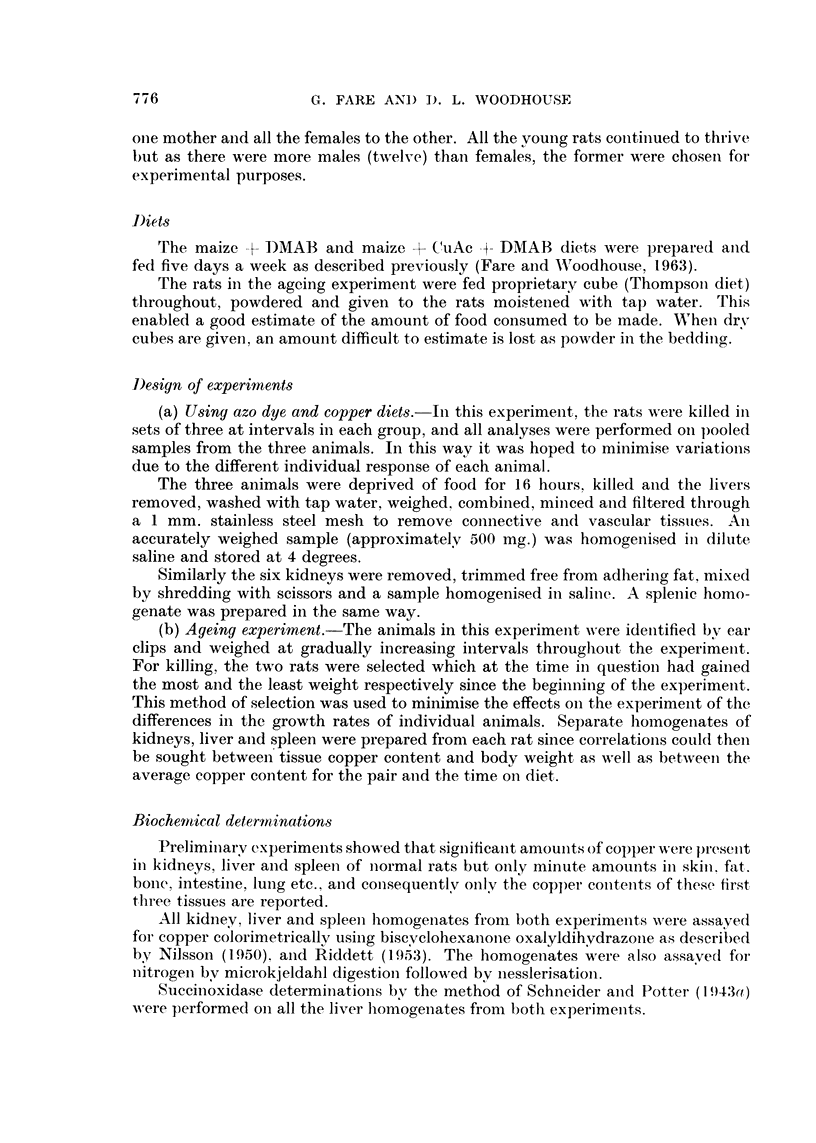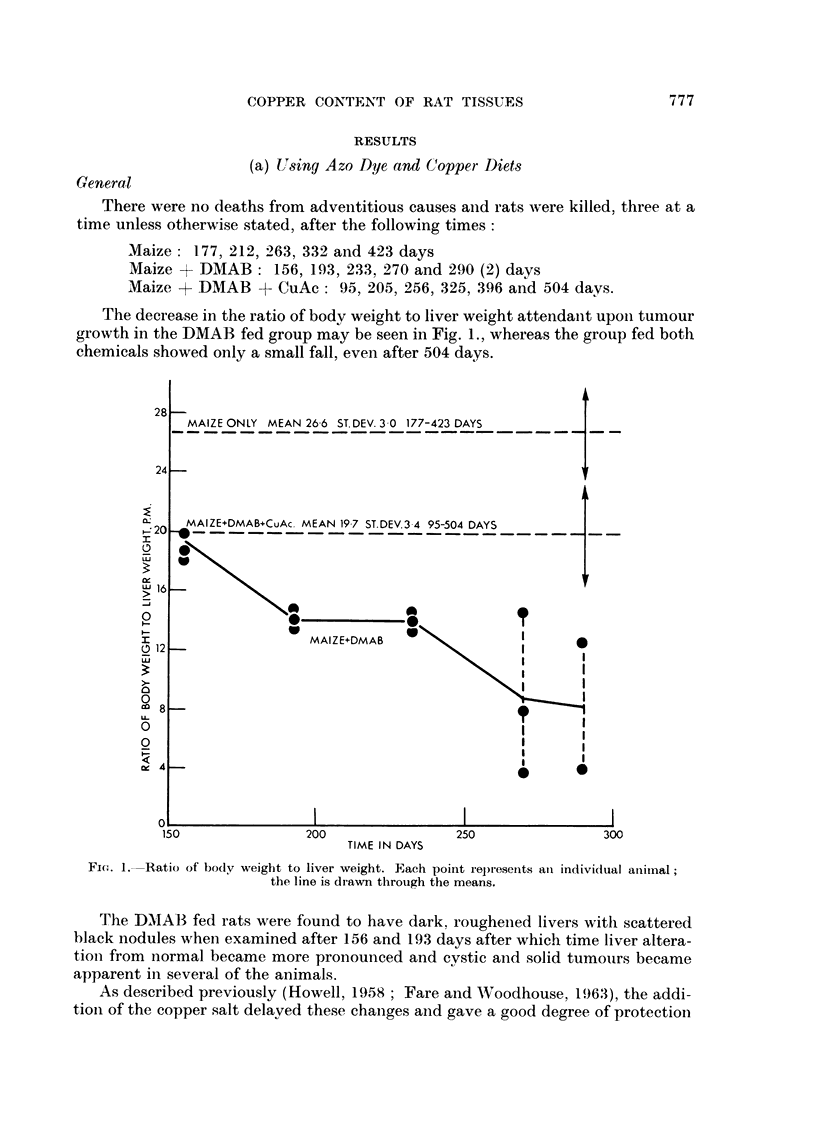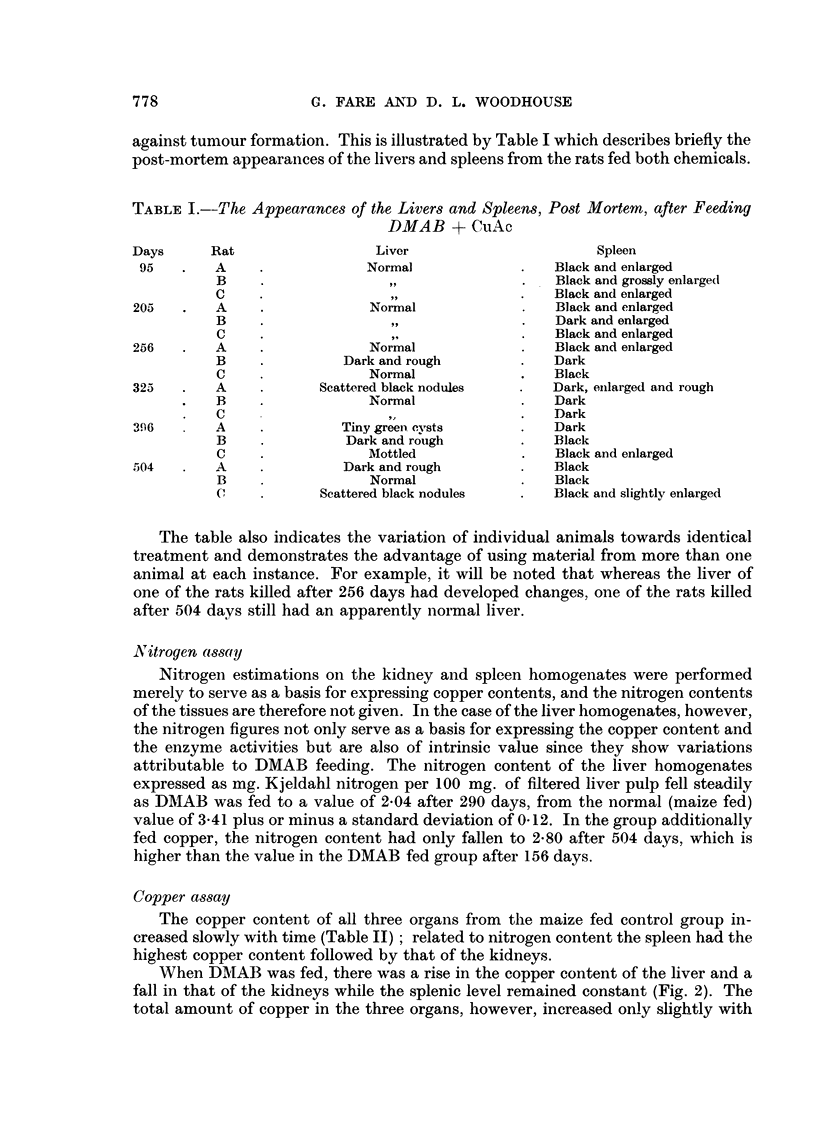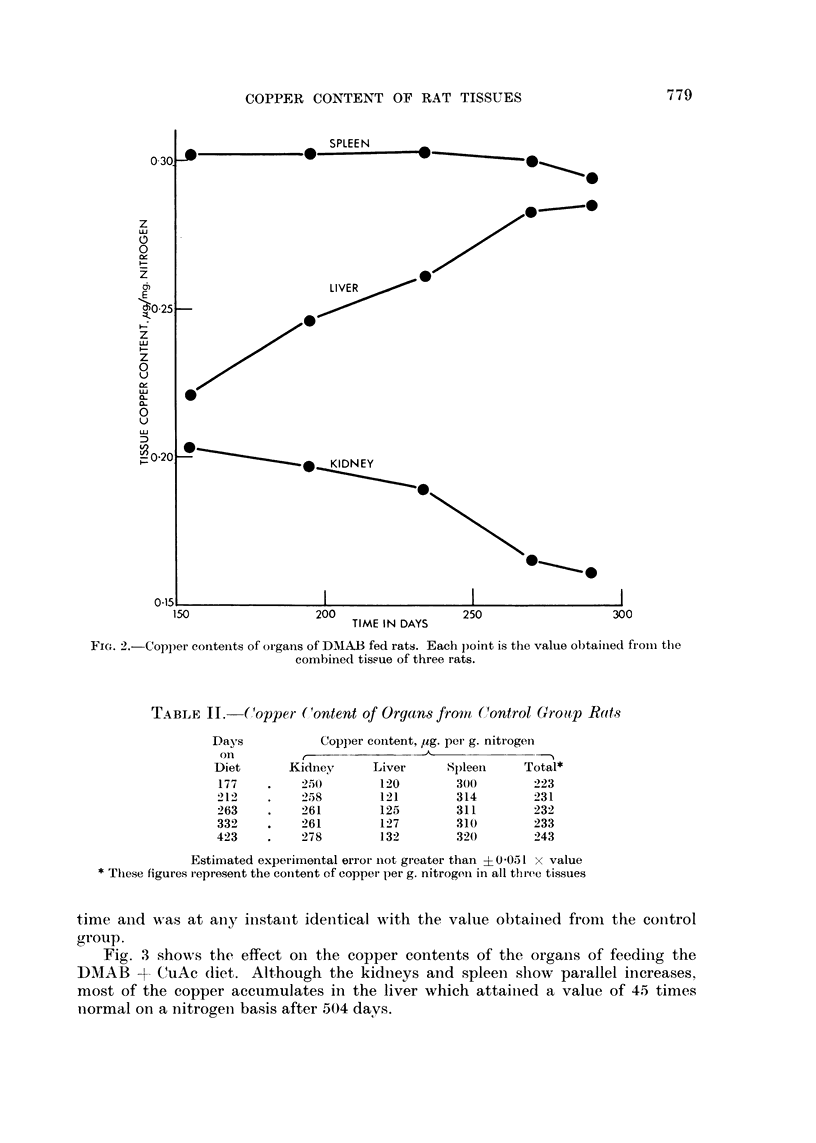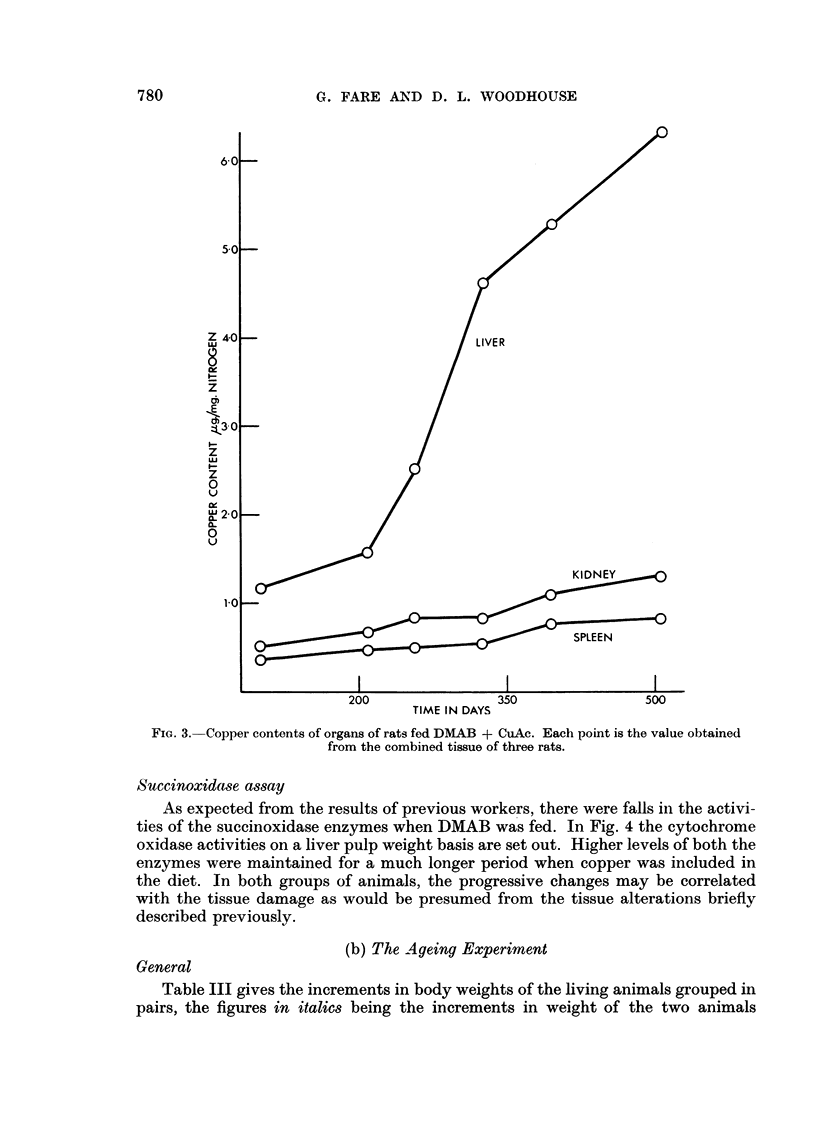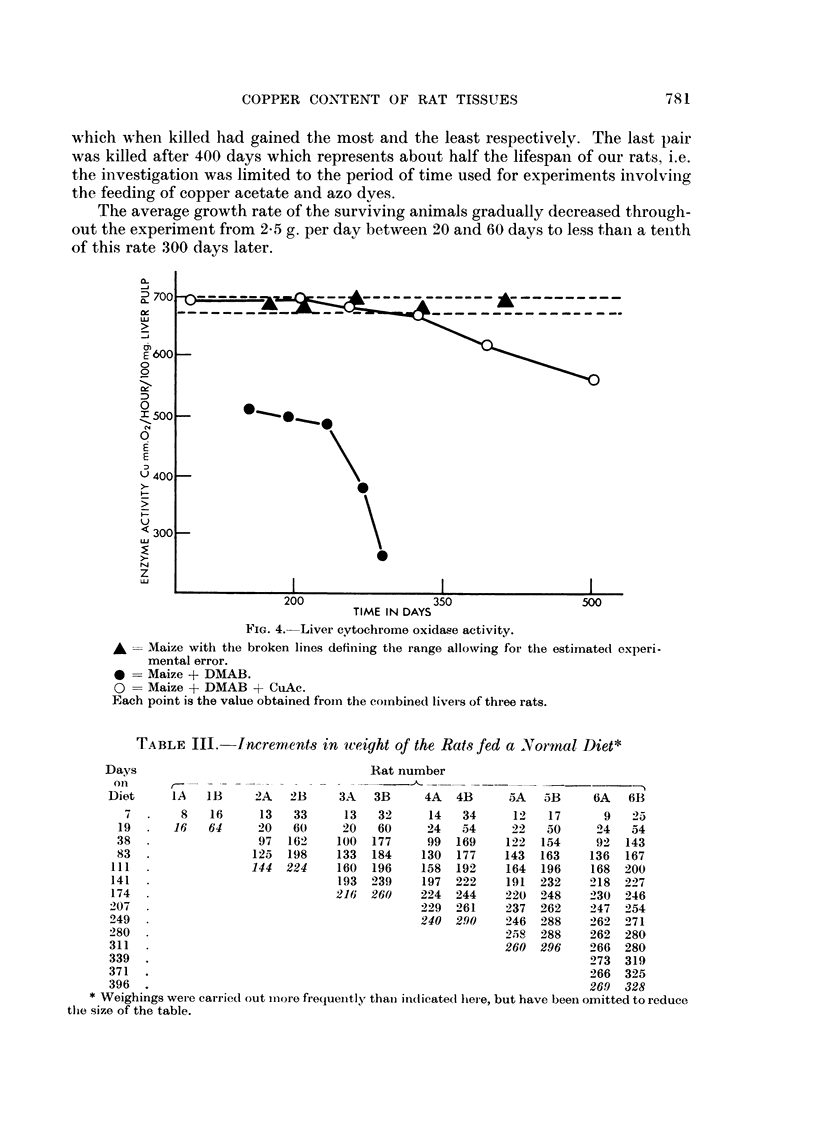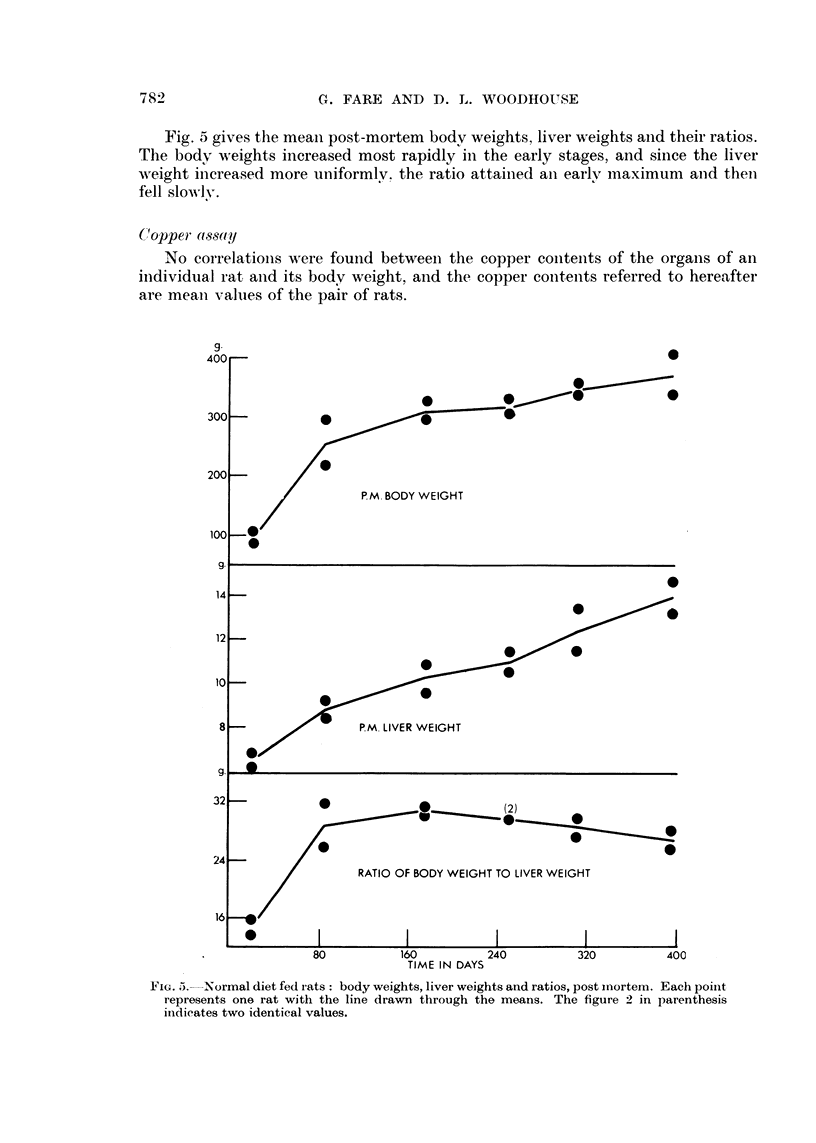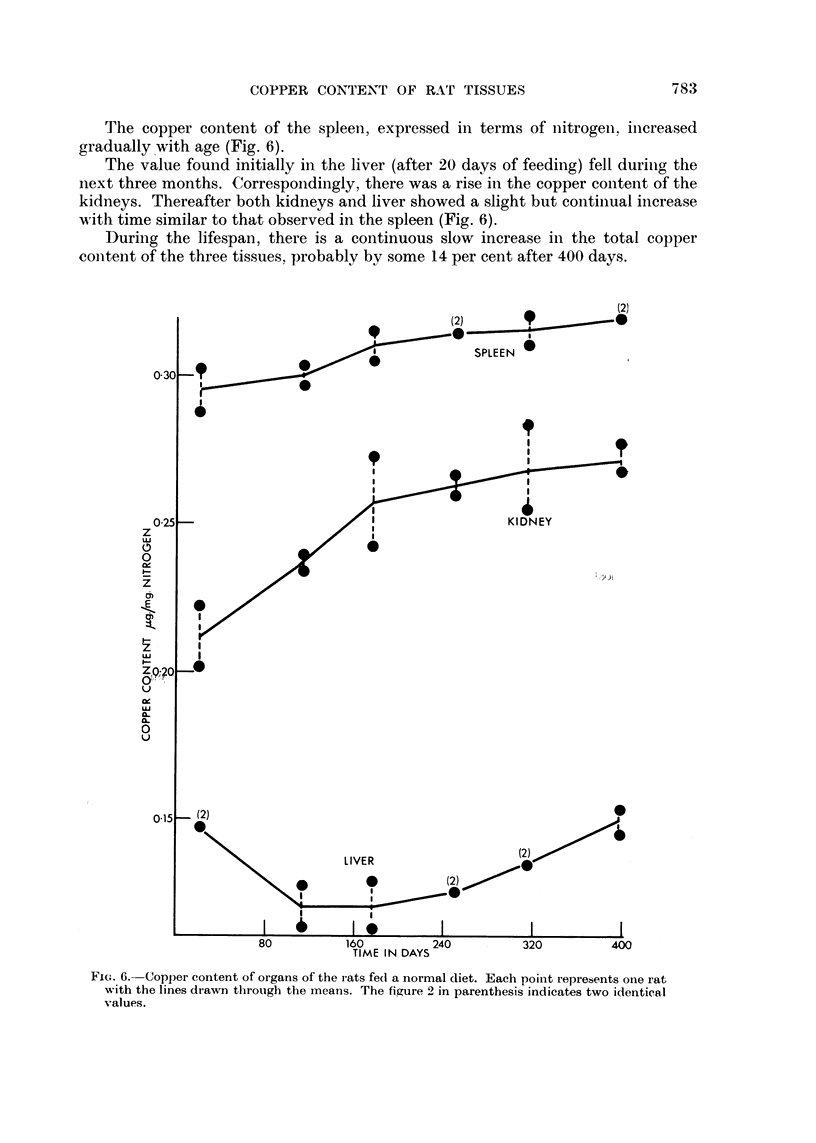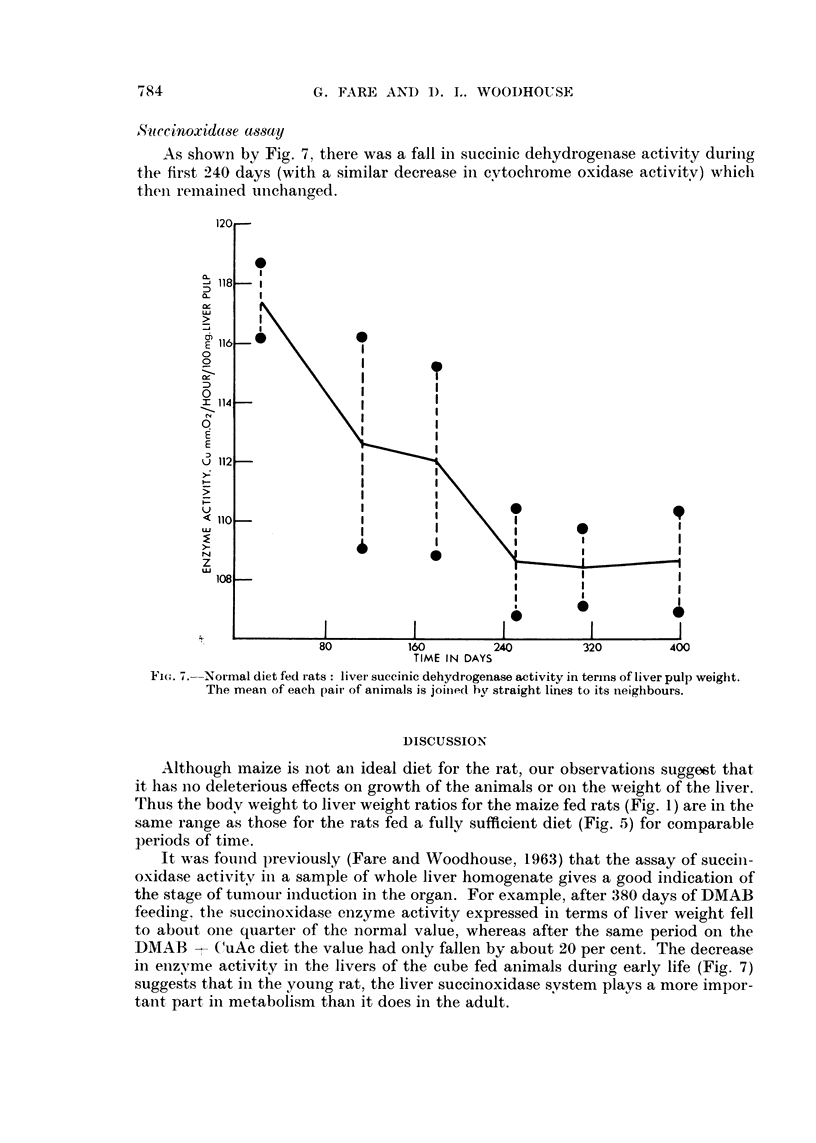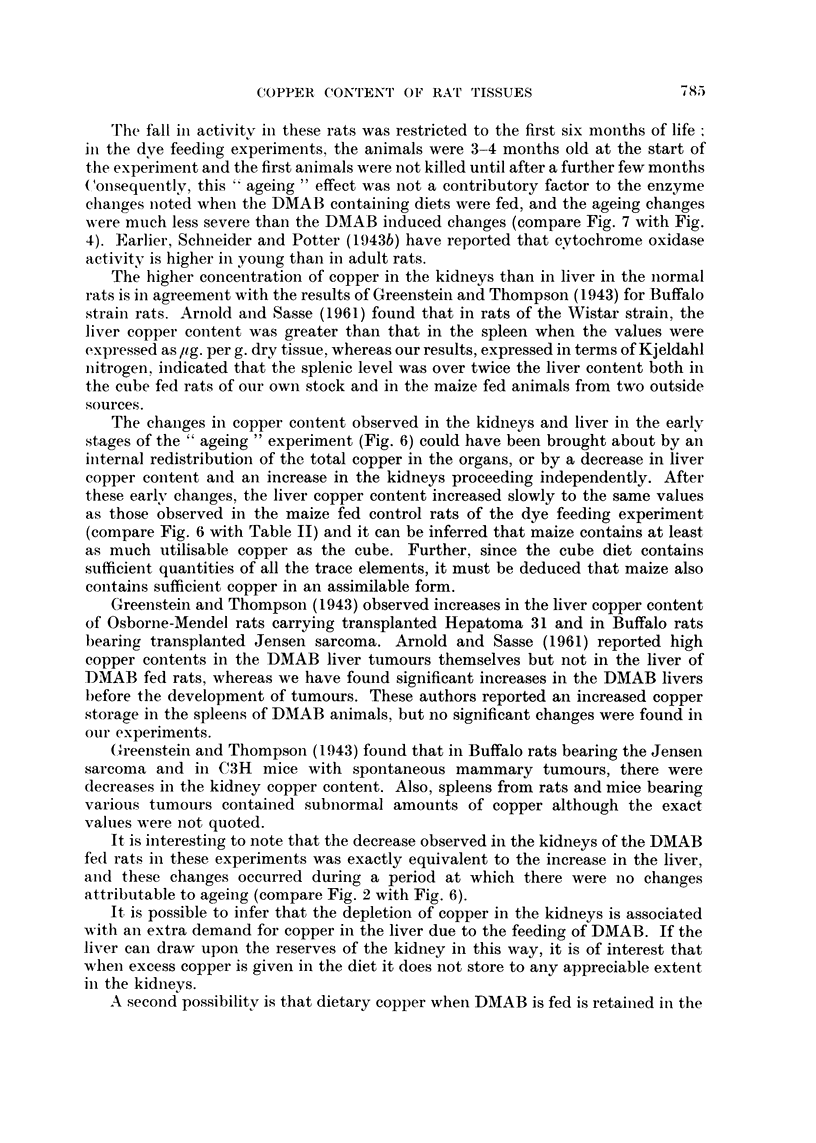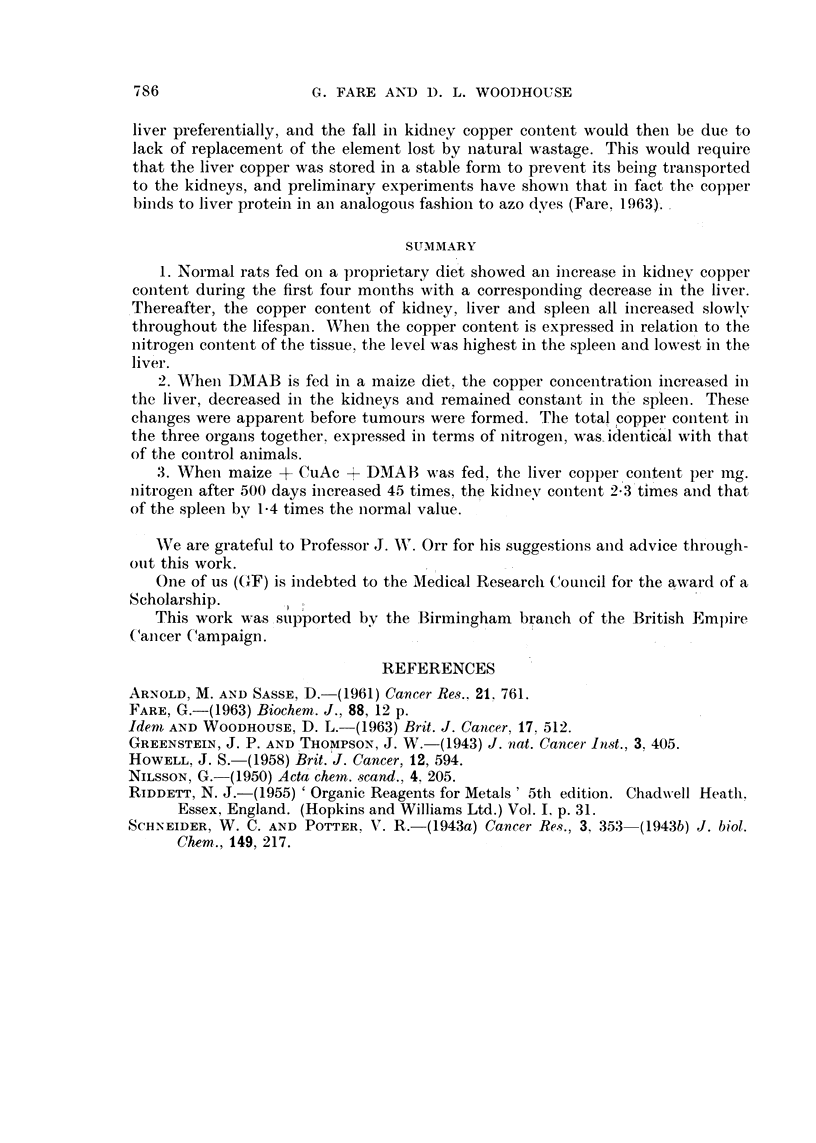# The Distribution of Copper in the Tissues of the Rat: The Effects of Age and of Feeding pDimethylaminoazobenzene with and without copper Acetate

**DOI:** 10.1038/bjc.1963.97

**Published:** 1963-12

**Authors:** G. Fare, D. L. Woodhouse


### THE DISTRIBUTION OF COPPER IN THE TISSUES OF THE RAT: THE EFFECTS OF AGE AND OF FEEDING *p*-DIMETHYLAMINO-AZOBENZENE WITH AND WITHOUT COPPER ACETATE

G. FARE AND D. L. WOODHOUSE

*From the Cancer Research Laboratories, Department of Pathology, Medical School, Birmingham* 15

Received for publication August 21, 1963

IT was found previously (Fare and Woodhouse, 1963) that when rats were fed *p*-dimethylaminoazobenzene (DMAB), there was a gradual increase in the concentration of copper in the liver, an increase of 35 per cent above normal being observed after 380 days on a diet of maize containing 0·09 per cent DMAB. We have since found a decrease in the copper content of the kidneys.

When 0·5 per cent of cupric oxyacetate hexahydrate is fed in addition to the DMAB, a good degree of protection is given against the development of liver tumours (Howell, 1958) and the copper content of the liver increases enormously to 40 times normal after 380 days (Fare and Woodhouse, 1963). The association of increased copper in the liver when excess copper is fed with inhibition of tumours, suggests that the alterations in copper content of kidney and liver when DMAB is fed alone play some part in a possible intrinsic defence mechanism against DMAB as a carcinogen.

This paper, therefore, describes the results of copper assays on rat tissues after feeding the carcinogen, alone and also together with copper for periods of up to 400 days, together with the values obtained from control rats fed maize only.

It was necessary to determine whether the changes in the DMAB-fed rats were partially dependent upon the age of the animals and upon the possibly low copper content of maize, and so a further control group of rats was fed a proprietary diet which contains all the necessary trace elements in sufficient amount and in an assimilable form.

### MATERIALS AND METHODS

### *Animals*

Twenty-five female albino rats were obtained from two outside sources. The two sets were distinguished by ear clips and were given an adequate diet until they were 4–5 months old. During this time, three animals died and the remaining 47 were taken at random and assigned to the three dietary groups. Fifteen rats formed the maize fed control group, 14 were fed maize +0·09 per cent DMAB and 18 were fed maize +0·09 per cent DMAB +0·5 per cent copper acetate (CuAc).

For the " ageing " experiment, two 3-day old litters of rats from our outbred laboratory stock were combined. The males from both litters were returned to the one mother and all the females to the other. All the young rats continued to thrive but as there were more males (twelve) than females, the former were chosen for experimental purposes.

### *Diets*

The maize + DMAB and maize + CuAc + DMAB diets were prepared and fed five days a week as described previously (Fare and Woodhouse, 1963).

The rats in the ageing experiment were fed proprietary cube (Thompson diet) throughout, powdered and given to the rats moistened with tap water. This enabled a good estimate of the amount of food consumed to be made. When dry cubes are given, an amount difficult to estimate is lost as powder in the bedding.

### *Design of experiments*

(a) *Using azo dye and copper diets.*—In this experiment, the rats were killed in sets of three at intervals in each group, and all analyses were performed on pooled samples from the three animals. In this way it was hoped to minimise variations due to the different individual response of each animal.

The three animals were deprived of food for 16 hours, killed and the livers removed, washed with tap water, weighed, combined, minced and filtered through a 1 mm. stainless steel mesh to remove connective and vascular tissues. An accurately weighed sample (approximately 500 mg.) was homogenised in dilute saline and stored at 4 degrees.

Similarly the six kidneys were removed, trimmed free from adhering fat, mixed by shredding with scissors and a sample homogenised in saline. A splenic homogenate was prepared in the same way.

(b) *Ageing experiment.*—The animals in this experiment were identified by ear clips and weighed at gradually increasing intervals throughout the experiment. For killing, the two rats were selected which at the time in question had gained the most and the least weight respectively since the beginning of the experiment. This method of selection was used to minimise the effects on the experiment of the differences in the growth rates of individual animals. Separate homogenates of kidneys, liver and spleen were prepared from each rat since correlations could then be sought between tissue copper content and body weight as well as between the average copper content for the pair and the time on diet.

### *Biochemical determinations*

Preliminary experiments showed that significant amounts of copper were present in kidneys, liver and spleen of normal rats but only minute amounts in skin, fat, bone, intestine, lung etc., and consequently only the copper contents of these first three tissues are reported.

All kidney, liver and spleen homogenates from both experiments were assayed for copper colorimetrically using biscyclohexanone oxalyldihydrazone as described by Nilsson (1950), and Riddett (1953). The homogenates were also assayed for nitrogen by microkjeldahl digestion followed by nesslerisation.

Succinoxidase determinations by the method of Schneider and Potter (1943*a*) were performed on all the liver homogenates from both experiments.

### RESULTS

### (a) *Using Azo Dye and Copper Diets*

*General*

There were no deaths from adventitious causes and rats were killed, three at a time unless otherwise stated, after the following times :

Maize : 177, 212, 263, 332 and 423 days
Maize + DMAB : 156, 193, 233, 270 and 290 (2) days
Maize + DMAB + CuAc : 95, 205, 256, 325, 396 and 504 days.

The decrease in the ratio of body weight to liver weight attendant upon tumour growth in the DMAB fed group may be seen in Fig. 1., whereas the group fed both chemicals showed only a small fall, even after 504 days.

FIG. 1.—Ratio of body weight to liver weight. Each point represents an individual animal ; the line is drawn through the means.

The DMAB fed rats were found to have dark, roughened livers with scattered black nodules when examined after 156 and 193 days after which time liver alteration from normal became more pronounced and cystic and solid tumours became apparent in several of the animals.

As described previously (Howell, 1958 ; Fare and Woodhouse, 1963), the addition of the copper salt delayed these changes and gave a good degree of protection against tumour formation. This is illustrated by Table I which describes briefly the post-mortem appearances of the livers and spleens from the rats fed both chemicals.

TABLE I.—*The Appearances of the Livers and Spleens, Post Mortem, after Feeding DMAB* + CuAc

| Days | Rat | Liver | Spleen |
|---|---|---|---|
| 95 | A | Normal | Black and enlarged |
| | B | ,, | Black and grossly enlarged |
| | C | ,, | Black and enlarged |
| 205 | A | Normal | Black and enlarged |
| | B | ,, | Dark and enlarged |
| | C | ,, | Black and enlarged |
| 256 | A | Normal | Black and enlarged |
| | B | Dark and rough | Dark |
| | C | Normal | Black |
| 325 | A | Scattered black nodules | Dark, enlarged and rough |
| | B | Normal | Dark |
| | C | ,, | Dark |
| 396 | A | Tiny green cysts | Dark |
| | B | Dark and rough | Black |
| | C | Mottled | Black and enlarged |
| 504 | A | Dark and rough | Black |
| | B | Normal | Black |
| | C | Scattered black nodules | Black and slightly enlarged |

The table also indicates the variation of individual animals towards identical treatment and demonstrates the advantage of using material from more than one animal at each instance. For example, it will be noted that whereas the liver of one of the rats killed after 256 days had developed changes, one of the rats killed after 504 days still had an apparently normal liver.

*Nitrogen assay*

Nitrogen estimations on the kidney and spleen homogenates were performed merely to serve as a basis for expressing copper contents, and the nitrogen contents of the tissues are therefore not given. In the case of the liver homogenates, however, the nitrogen figures not only serve as a basis for expressing the copper content and the enzyme activities but are also of intrinsic value since they show variations attributable to DMAB feeding. The nitrogen content of the liver homogenates expressed as mg. Kjeldahl nitrogen per 100 mg. of filtered liver pulp fell steadily as DMAB was fed to a value of 2·04 after 290 days, from the normal (maize fed) value of 3·41 plus or minus a standard deviation of 0·12. In the group additionally fed copper, the nitrogen content had only fallen to 2·80 after 504 days, which is higher than the value in the DMAB fed group after 156 days.

*Copper assay*

The copper content of all three organs from the maize fed control group increased slowly with time (Table II); related to nitrogen content the spleen had the highest copper content followed by that of the kidneys.

When DMAB was fed, there was a rise in the copper content of the liver and a fall in that of the kidneys while the splenic level remained constant (Fig. 2). The total amount of copper in the three organs, however, increased only slightly with

FIG. 2.—Copper contents of organs of DMAB fed rats. Each point is the value obtained from the combined tissue of three rats.

TABLE II.—*Copper Content of Organs from Control Group Rats*

| Days on Diet | Copper content, µg. per g. nitrogen | | | |
|---|---|---|---|---|
| | Kidney | Liver | Spleen | Total* |
| 177 . | 250 | 120 | 300 | 223 |
| 212 . | 258 | 121 | 314 | 231 |
| 263 . | 261 | 125 | 311 | 232 |
| 332 . | 261 | 127 | 310 | 233 |
| 423 . | 278 | 132 | 320 | 243 |

Estimated experimental error not greater than $\pm 0{\cdot}051 \times$ value
* These figures represent the content of copper per g. nitrogen in all three tissues time and was at any instant identical with the value obtained from the control group.

Fig. 3 shows the effect on the copper contents of the organs of feeding the DMAB + CuAc diet. Although the kidneys and spleen show parallel increases, most of the copper accumulates in the liver which attained a value of 45 times normal on a nitrogen basis after 504 days.

FIG. 3.—Copper contents of organs of rats fed DMAB + CuAc. Each point is the value obtained from the combined tissue of three rats.

*Succinoxidase assay*

As expected from the results of previous workers, there were falls in the activities of the succinoxidase enzymes when DMAB was fed. In Fig. 4 the cytochrome oxidase activities on a liver pulp weight basis are set out. Higher levels of both the enzymes were maintained for a much longer period when copper was included in the diet. In both groups of animals, the progressive changes may be correlated with the tissue damage as would be presumed from the tissue alterations briefly described previously.

(b) *The Ageing Experiment*

*General*

Table III gives the increments in body weights of the living animals grouped in pairs, the figures *in italics* being the increments in weight of the two animals which when killed had gained the most and the least respectively. The last pair was killed after 400 days which represents about half the lifespan of our rats, i.e. the investigation was limited to the period of time used for experiments involving the feeding of copper acetate and azo dyes.

The average growth rate of the surviving animals gradually decreased throughout the experiment from 2·5 g. per day between 20 and 60 days to less than a tenth of this rate 300 days later.

Fig. 4.—Liver cytochrome oxidase activity.

▲ = Maize with the broken lines defining the range allowing for the estimated experimental error.
● = Maize + DMAB.
○ = Maize + DMAB + CuAc.
Each point is the value obtained from the combined livers of three rats.

Table III.—*Increments in weight of the Rats fed a Normal Diet**

| Days on Diet | Rat number | | | | | | | | | | | |
|---|---|---|---|---|---|---|---|---|---|---|---|---|
| | 1A | 1B | 2A | 2B | 3A | 3B | 4A | 4B | 5A | 5B | 6A | 6B |
| 7 . | 8 | 16 | 13 | 33 | 13 | 32 | 14 | 34 | 12 | 17 | 9 | 25 |
| 19 . | *16* | *64* | 20 | 60 | 20 | 60 | 24 | 54 | 22 | 50 | 24 | 54 |
| 38 . | | | 97 | 162 | 100 | 177 | 99 | 169 | 122 | 154 | 92 | 143 |
| 83 . | | | 125 | 198 | 133 | 184 | 130 | 177 | 143 | 163 | 136 | 167 |
| 111 . | | | *144* | *224* | 160 | 196 | 158 | 192 | 164 | 196 | 168 | 200 |
| 141 . | | | | | 193 | 239 | 197 | 222 | 191 | 232 | 218 | 227 |
| 174 . | | | | | *216* | *260* | 224 | 244 | 220 | 248 | 230 | 246 |
| 207 . | | | | | | | 229 | 261 | 237 | 262 | 247 | 254 |
| 249 . | | | | | | | *240* | *290* | 246 | 288 | 262 | 271 |
| 280 . | | | | | | | | | 258 | 288 | 262 | 280 |
| 311 . | | | | | | | | | *260* | *296* | 266 | 280 |
| 339 . | | | | | | | | | | | 273 | 319 |
| 371 . | | | | | | | | | | | 266 | 325 |
| 396 . | | | | | | | | | | | *269* | *328* |

* Weighings were carried out more frequently than indicated here, but have been omitted to reduce the size of the table.

Fig. 5 gives the mean post-mortem body weights, liver weights and their ratios. The body weights increased most rapidly in the early stages, and since the liver weight increased more uniformly, the ratio attained an early maximum and then fell slowly.

*Copper assay*

No correlations were found between the copper contents of the organs of an individual rat and its body weight, and the copper contents referred to hereafter are mean values of the pair of rats.

FIG. 5.—Normal diet fed rats : body weights, liver weights and ratios, post mortem. Each point represents one rat with the line drawn through the means. The figure 2 in parenthesis indicates two identical values.

The copper content of the spleen, expressed in terms of nitrogen, increased gradually with age (Fig. 6).

The value found initially in the liver (after 20 days of feeding) fell during the next three months. Correspondingly, there was a rise in the copper content of the kidneys. Thereafter both kidneys and liver showed a slight but continual increase with time similar to that observed in the spleen (Fig. 6).

During the lifespan, there is a continuous slow increase in the total copper content of the three tissues, probably by some 14 per cent after 400 days.

FIG. 6.—Copper content of organs of the rats fed a normal diet. Each point represents one rat with the lines drawn through the means. The figure 2 in parenthesis indicates two identical values.

*Succinoxidase assay*

As shown by Fig. 7, there was a fall in succinic dehydrogenase activity during the first 240 days (with a similar decrease in cytochrome oxidase activity) which then remained unchanged.

FIG. 7.—Normal diet fed rats : liver succinic dehydrogenase activity in terms of liver pulp weight. The mean of each pair of animals is joined by straight lines to its neighbours.

### DISCUSSION

Although maize is not an ideal diet for the rat, our observations suggest that it has no deleterious effects on growth of the animals or on the weight of the liver. Thus the body weight to liver weight ratios for the maize fed rats (Fig. 1) are in the same range as those for the rats fed a fully sufficient diet (Fig. 5) for comparable periods of time.

It was found previously (Fare and Woodhouse, 1963) that the assay of succinoxidase activity in a sample of whole liver homogenate gives a good indication of the stage of tumour induction in the organ. For example, after 380 days of DMAB feeding, the succinoxidase enzyme activity expressed in terms of liver weight fell to about one quarter of the normal value, whereas after the same period on the DMAB + CuAc diet the value had only fallen by about 20 per cent. The decrease in enzyme activity in the livers of the cube fed animals during early life (Fig. 7) suggests that in the young rat, the liver succinoxidase system plays a more important part in metabolism than it does in the adult.

The fall in activity in these rats was restricted to the first six months of life ; in the dye feeding experiments, the animals were 3–4 months old at the start of the experiment and the first animals were not killed until after a further few months Consequently, this " ageing " effect was not a contributory factor to the enzyme changes noted when the DMAB containing diets were fed, and the ageing changes were much less severe than the DMAB induced changes (compare Fig. 7 with Fig. 4). Earlier, Schneider and Potter (1943*b*) have reported that cytochrome oxidase activity is higher in young than in adult rats.

The higher concentration of copper in the kidneys than in liver in the normal rats is in agreement with the results of Greenstein and Thompson (1943) for Buffalo strain rats. Arnold and Sasse (1961) found that in rats of the Wistar strain, the liver copper content was greater than that in the spleen when the values were expressed as $\mu$g. per g. dry tissue, whereas our results, expressed in terms of Kjeldahl nitrogen, indicated that the splenic level was over twice the liver content both in the cube fed rats of our own stock and in the maize fed animals from two outside sources.

The changes in copper content observed in the kidneys and liver in the early stages of the " ageing " experiment (Fig. 6) could have been brought about by an internal redistribution of the total copper in the organs, or by a decrease in liver copper content and an increase in the kidneys proceeding independently. After these early changes, the liver copper content increased slowly to the same values as those observed in the maize fed control rats of the dye feeding experiment (compare Fig. 6 with Table II) and it can be inferred that maize contains at least as much utilisable copper as the cube. Further, since the cube diet contains sufficient quantities of all the trace elements, it must be deduced that maize also contains sufficient copper in an assimilable form.

Greenstein and Thompson (1943) observed increases in the liver copper content of Osborne-Mendel rats carrying transplanted Hepatoma 31 and in Buffalo rats bearing transplanted Jensen sarcoma. Arnold and Sasse (1961) reported high copper contents in the DMAB liver tumours themselves but not in the liver of DMAB fed rats, whereas we have found significant increases in the DMAB livers before the development of tumours. These authors reported an increased copper storage in the spleens of DMAB animals, but no significant changes were found in our experiments.

Greenstein and Thompson (1943) found that in Buffalo rats bearing the Jensen sarcoma and in C3H mice with spontaneous mammary tumours, there were decreases in the kidney copper content. Also, spleens from rats and mice bearing various tumours contained subnormal amounts of copper although the exact values were not quoted.

It is interesting to note that the decrease observed in the kidneys of the DMAB fed rats in these experiments was exactly equivalent to the increase in the liver, and these changes occurred during a period at which there were no changes attributable to ageing (compare Fig. 2 with Fig. 6).

It is possible to infer that the depletion of copper in the kidneys is associated with an extra demand for copper in the liver due to the feeding of DMAB. If the liver can draw upon the reserves of the kidney in this way, it is of interest that when excess copper is given in the diet it does not store to any appreciable extent in the kidneys.

A second possibility is that dietary copper when DMAB is fed is retained in the liver preferentially, and the fall in kidney copper content would then be due to lack of replacement of the element lost by natural wastage. This would require that the liver copper was stored in a stable form to prevent its being transported to the kidneys, and preliminary experiments have shown that in fact the copper binds to liver protein in an analogous fashion to azo dyes (Fare, 1963).

SUMMARY

1. Normal rats fed on a proprietary diet showed an increase in kidney copper content during the first four months with a corresponding decrease in the liver. Thereafter, the copper content of kidney, liver and spleen all increased slowly throughout the lifespan. When the copper content is expressed in relation to the nitrogen content of the tissue, the level was highest in the spleen and lowest in the liver.

2. When DMAB is fed in a maize diet, the copper concentration increased in the liver, decreased in the kidneys and remained constant in the spleen. These changes were apparent before tumours were formed. The total copper content in the three organs together, expressed in terms of nitrogen, was identical with that of the control animals.

3. When maize + CuAc + DMAB was fed, the liver copper content per mg. nitrogen after 500 days increased 45 times, the kidney content 2·3 times and that of the spleen by 1·4 times the normal value.

We are grateful to Professor J. W. Orr for his suggestions and advice throughout this work.

One of us (GF) is indebted to the Medical Research Council for the award of a Scholarship.

This work was supported by the Birmingham branch of the British Empire Cancer Campaign.

### REFERENCES

Arnold, M. and Sasse, D.—(1961) *Cancer Res.*, **21**, 761.

Fare, G.—(1963) *Biochem. J.*, **88**, 12 p.

*Idem* and Woodhouse, D. L.—(1963) *Brit. J. Cancer*, **17**, 512.

Greenstein, J. P. and Thompson, J. W.—(1943) *J. nat. Cancer Inst.*, **3**, 405.

Howell, J. S.—(1958) *Brit. J. Cancer*, **12**, 594.

Nilsson, G.—(1950) *Acta chem. scand.*, **4**, 205.

Riddett, N. J.—(1955) ' Organic Reagents for Metals ' 5th edition. Chadwell Heath, Essex, England. (Hopkins and Williams Ltd.) Vol. I, p. 31.

Schneider, W. C. and Potter, V. R.—(1943*a*) *Cancer Res.*, **3**, 353—(1943*b*) *J. biol. Chem.*, **149**, 217.